# Data rescue: saving environmental data from extinction

**DOI:** 10.1098/rspb.2022.0938

**Published:** 2022-07-27

**Authors:** Ellen K. Bledsoe, Joseph B. Burant, Gracielle T. Higino, Dominique G. Roche, Sandra A. Binning, Kerri Finlay, Jason Pither, Laura S. Pollock, Jennifer M. Sunday, Diane S. Srivastava

**Affiliations:** ^1^ The Living Data Project, Canadian Institute of Ecology and Evolution, Vancouver, British Columbia, Canada; ^2^ School of Natural Resources and the Environment, University of Arizona, Tucson, AZ, USA; ^3^ Department of Biology, University of Regina, Regina, Saskatchewan, Canada; ^4^ Department of Biology, McGill University, Montreal, Quebec, Canada; ^5^ Département de sciences biologiques, Université de Montréal, Montréal, Québec, Canada; ^6^ Department of Zoology and Biodiversity Research Centre, University of British Columbia, Vancouver, British Columbia, Canada; ^7^ Department of Biology and Institute for Environment & Interdisciplinary Science, Carleton University, Ottawa, Ontario, Canada; ^8^ Department of Biology and Okanagan Institute for Biodiversity, Resilience, and Ecosystem Services, University of British Columbia, Kelowna, British Columbia, Canada

**Keywords:** data archiving, historical data, long-term ecological data, open science, reproducibility, transparency

## Abstract

Historical and long-term environmental datasets are imperative to understanding how natural systems respond to our changing world. Although immensely valuable, these data are at risk of being lost unless actively curated and archived in data repositories. The practice of data rescue, which we define as identifying, preserving, and sharing valuable data and associated metadata at risk of loss, is an important means of ensuring the long-term viability and accessibility of such datasets. Improvements in policies and best practices around data management will hopefully limit future need for data rescue; these changes, however, do not apply retroactively. While rescuing data is not new, the term lacks formal definition, is often conflated with other terms (i.e. data reuse), and lacks general recommendations. Here, we outline seven key guidelines for effective rescue of historically collected and unmanaged datasets. We discuss prioritization of datasets to rescue, forming effective data rescue teams, preparing the data and associated metadata, and archiving and sharing the rescued materials. In an era of rapid environmental change, the best policy solutions will require evidence from both contemporary and historical sources. It is, therefore, imperative that we identify and preserve valuable, at-risk environmental data before they are lost to science.

## 1. Why rescue data?

Data are among the most valuable outputs of research and scholarship; beyond helping answer important questions, they inform new lines of inquiry, new testable hypotheses and future data collection efforts. Observational and experimental data derived from ecology, evolution, conservation, and environmental sciences (hereafter, environmental data) are essential to establishing historical trajectories of ecosystems (baselines) [1], understanding how species and communities respond to environmental change [2] and designing and evaluating the outcomes of management efforts [3]. While data collection is often targeted to particular populations, communities or locations, the reuse (i.e. aggregation, collation and synthesis) of data from different contexts is essential to establishing broader ecological knowledge and informing conservation management [4]. Yet, despite their substantial value, data are often misplaced, filed away or otherwise rendered unusable, often through poor data management practices [5]. In their unusable and 'at-risk' state, these data represent an egregious waste of resources expended on their collection (box 1) [8]. Languishing data, however, also offer an enormous opportunity. *Data rescue*—defined here as the identification, preservation and sharing of valuable data and associated metadata at risk of loss—has the potential to realize substantial benefits for society, especially considering the crucial roles that baseline data play in informing management and policy decisions. The ultimate goal of data rescue is to make previously inaccessible or poorly preserved data available for (re)use, ideally through archiving them in a permanent, publicly accessible and reusable format.

Box 1. **Spilt oil, spent money and lost data.**

In 1989, the oil tanker *Exxon Valdez* struck the Bligh Reef in Prince William Sound, less than 2.5 km from the Alaskan shore. As a result, approximately 37 000 tonnes of crude oil spilled into the sound, leading to catastrophic short- and long-term ecological consequences. The *Exxon Valdez* Oil Spill Trustee Council (EVOSTC) was established in 1991 to oversee the spending of funds from a civil settlement in 1991 between *Exxon,* the United States federal government and the state government of Alaska. A large portion of funds were directed towards determining and monitoring the impacts of the oil spill on oceanographic, environmental and ecological conditions. Prior to 2003, there was no requirement for data preservation or availability; afterwards, all projects were awarded under explicit conditions from EVOSTC that data be preserved and made publicly available [6]. In their annual report from 2010, the EVOSTC notes that some $151.2 million USD were spent on “research, monitoring and general restoration” during 1992–2010 fiscal years [7].

From 2012 to 2014, a group of researchers from the National Center for Ecological Analysis and Synthesis worked to recover the historical datasets funded by EVOSTC, focusing specifically on data collected between 1989 and 2010 [6]. Of the 419 projects funded by EVOSTC during this time, only 27% of the datasets were able to be recovered; after a total of 5 years hunting down datasets, this grew to 30% [6].

Using these numbers, we can roughly estimate the money spent on research for which the data are unrecoverable (70% of datasets): *approximately $105 million USD was spent collecting data that are no longer recoverable and, therefore, effectively non-existent to science*. While we do not know the distribution of years from which data were recovered or how money was allocated by year, this is probably a conservative estimate given that the original cost does not include the first 3 years following the spill, when extensive ecological assessments would have been completed.

Data rescue is particularly important in the environmental sciences for three reasons. First, because environmental processes are context-dependent, they often have historical components. Such records are essential in understanding the trajectory of environmental change and guiding policy to mitigate or adapt to this change [9]. For example, information obtained by rescuing salmon samples collected in the early twentieth century dramatically changed our understanding of how salmon stocks have declined over the last century [10]. Second, environmental datasets are often small and local, constrained by both organismal-level data collection and the fine spatial scale of many of the underlying processes. Therefore, to obtain powerful tests of theory and the generality of mechanisms across heterogeneity in ecosystems and species, we need to synthesize across datasets; saving data is essential for synthesis. Third, there has been a computational revolution in the types of analyses we can do and the amount of data that can be included [11]. This means that we can now finally perform powerful analyses of some of the exquisitely detailed data collected before the information revolution.

In recent years, there has also been a strong push from within scientific and scholarly communities for increased openness in science, including ecology and evolution (e.g. [12]). Calls for more transparency and accessibility in science are not new (e.g. [13]), although the last decade has seen a surge in general awareness and promotion of open science practices (e.g. open access publishing and open data, code, software and peer-review) and their benefits [14]. These initiatives have not been without criticism, with many researchers unsure about sharing their data owing to real or perceived concerns about data misuse and loss of control [15–17]. Others have acknowledged important caveats to the general appeal for openness (e.g. considerations about security, confidentiality, equity and Indigenous data sovereignty and governance; [18–21]). Despite the legitimacy of (some of) these concerns, the benefits of data sharing are apparent [14,22]. Even so, large amounts of data remain private and unavailable for reuse. For example, in a sample of greater than 4000 ecology and evolution papers, only one in five papers (21.5%) had a data availability statement or associated open data [23], and less than half of archived datasets in ecology and evolution are reusable [23,24]).

Open science initiatives have developed rapidly, and the number of institutions, governments, funding agencies and publishers who have implemented policies that require the open, permanent, and accessible sharing of data is increasing (e.g. FAIR data principles [25], the Ecological Society of America's new Open Research policy and the European Commission's OpenAIRE open access and open data policy). These requirements, and participation by scientists, will enhance our ability to evaluate, re-use and synthesize increasingly rich and complex ecological data. However, open data policies are not retroactive and, therefore, do little to address issues of access to and preservation of previously collected data [5]. Arguably, data collected prior to the adoption of widespread sharing practices remain a public good, funded by taxpayers and governments, so rescuing datasets to ensure their longevity and accessibility is imperative.

Here, we present general guidelines for implementing data rescue, with a focus on environmental data. These recommendations are based on past and ongoing data rescue efforts by the Living Data Project, an initiative of the Canadian Institute of Ecology and Evolution (CIEE), which aims to identify and secure vulnerable datasets and bring new life to them through collaborative analysis and synthesis (box 2). We hope these guidelines will: (i) focus attention on the current threats to the usability and integrity of previously collected data, (ii) stimulate broader consideration of the use of historical datasets for current research efforts, (iii) encourage people with knowledge of unarchived data to preserve them, (iv) provide a reference for those looking to apply data rescue techniques either *ad hoc* or as part of a broader initiative, and (v) help foster a strong culture of data stewardship such that data rescue becomes unnecessary in the future.

Box 2. **Data rescue examples from the Living Data Project.**

**Seeing the forest data for the trees**

Upon the retirement or death of a professor, students or colleagues sometimes must take the reins and piece together documents and data from decades-old research projects.

*Step 1 (Data prioritization)*: Dr George H. La Roi was a professor of forest ecology at the University of Alberta (UofA) for 35 years. Upon his passing, La Roi's children bequeathed his legacy of highly valuable data to his former colleague who had earlier taken over sampling some of his long-term plots. With no living data creator and the data in unorganized boxes containing unsorted datasheets, documents, CD-ROMs and picture slides (box 2.1), the data were at high risk of loss.

*Step 2 (Team creation)*: two of Dr La Roi's colleagues served as data stewards. Two graduate interns worked as data management experts, along with several undergraduate data entry technicians who sorted, entered and digitized the data.

*Step 3 (Metadata creation)*: thankfully, one of the loose files was a report with methodology for many of the data collection events. Initially, inventory on the data needed to be done. Finalized metadata were written and consolidated into one document for future reuse; while most of the data had clear documentation, some data were lost because of undetermined variable definitions and units.

*Step 4 (Data transfer and compilation)*: the boxes of data were sent to the graduate students and digitized data was transferred via a cloud-based service. The interns recovered data recorded at two different locations, both of which included similar measurements from plants. Some data were stored as printed scans of hand-filled datasheets and thus required digitization. Other data, which had already been entered and digitized, were stored in hundreds of text files which required extensive reformatting before they could be compiled into tidy, usable datasets.

*Step 5 (Data cleaning and validation)*: standard data cleaning and validation procedures were conducted, such as removing character values in numeric columns, checking the data for obvious outliers, etc. Extensive work was done to ensure consistent taxonomy throughout the decades of data collection.

*Step 6 (Data archiving)*: the data and metadata of this expansive dataset has been archived and made publicly available through UofA's Dataverse repository [26] with a CC-BY licence.

*Step 7 (Data sharing)*: all files associated with the data follow FAIR data guidelines, with extensive metadata, files in non-proprietary file formats, and uploaded to an open data repository with a DOI.

**Box 2.1.**
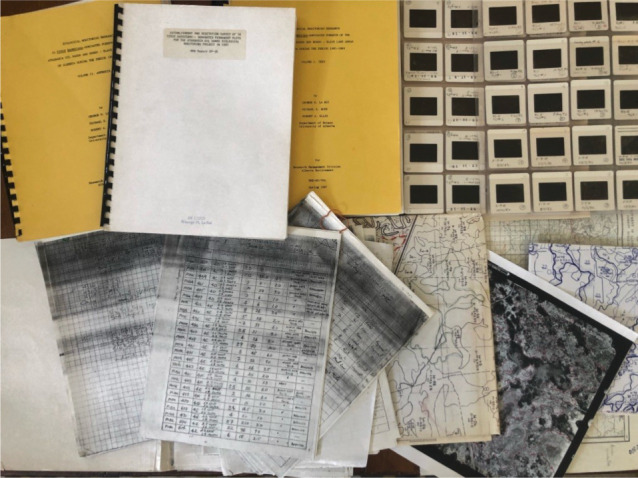
Photograph of loose data sheets, maps, reports and picture slides; these items and many more filled the boxes of research material left behind by Dr La Roi. Image credit: A. Hesketh.

**Out of the archives and into the (digital) light of day**

Theses and dissertations of former graduate students represent a rich source of historical data. In particular, those prepared prior to the advent of modern computer technologies and software (e.g. word processors) may contain troves of raw and summary data that remain undigitized.

*Step 1 (Data prioritization)*: this project was focused on securing the data contained in three, historical graduate theses from the University of British Columbia (UBC). While the specific questions and research topics differed, all three surveyed bird abundances in the same (or nearby) sites in Greater Vancouver, British Columbia, and combined present an opportunity to establish a baseline against which to compare current and future trends (electronic supplementary material, Box 2.S1). These data were prioritized because they were both at-risk (much of the data existed only in non-digital formats and none of the datasets are in active use) and deemed of high value (the data provide a valuable frame of reference for studying changes in urban bird diversity).

*Step 2 (Team creation)*: the project was proposed by a graduate student at UBC and was carried out in collaboration with a data rescue intern. As with the previous case, the original data creators were not directly involved in the data rescue, although one individual did provide a digital copy of the data contained within their thesis.

*Step 3 (Metadata creation)*: given the extensive data manipulation required, clear metadata were developed to document the various steps taken to generate the final datasets and document other details from the theses that were not captured during the digitization process.

*Step 4 (Data transfer and compilation)*: the intern first worked to transcribe and digitize the data from the two earlier theses, which were only available from the thesis repository as scans of typewritten documents. Among other challenges, digitization required the conversion of non-standard data types (box 2.2) into "tidy" forms that could be interpreted programmatically. Data from the third thesis [27] were made available by the original author in a spreadsheet and so only required cleaning, manipulation, and conversion to a non-proprietary format.

*Step 5 (Data cleaning and validation)*: later work included efforts to rationalize the datasets so they might be used in combination with each other (e.g. standardizing column names and combining similar tables into a single file).

*Step 6 (Data archiving)*: the data have been archived on the UBC Scholars Portal Dataverse repository [27–29] and cross-linked to the original theses.

*Step 7 (Data sharing)*: the datasets have been archived following FAIR principles, include detailed metadata describing the data rescue process, use non-proprietary file formats and have permanent DOIs.

**Box 2.2.**
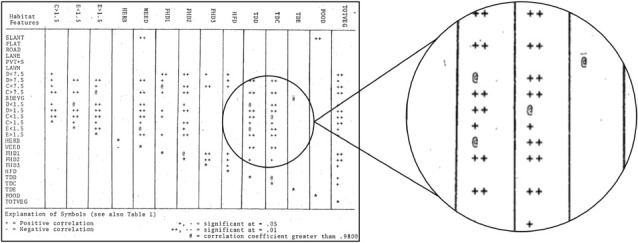
Example of non-standard data to be rationalized and digitized, representing the significance of correlations between habitat features. These symbols were converted to numeric factors during digitization. Reproduced with modification from Lancaster [30, see Appendix 4, p. 103–104 therein].

## 2. Guidelines for data rescue

Imperilled data can be found nearly everywhere (e.g. electronic supplementary material, box S1), such as non-profit organizations, conservation councils, academic institutions and government agencies (think: historical data only available on paper records or digitized data stored only on floppy disks). Although data to be rescued are plentiful, discoverability is challenged by the very fact that they have not yet been archived and, thus, are unfindable or inaccessible [25]. In ecology, for example, these issues lead to a low number of available datasets [23,31] and limit our capacity for knowledge synthesis. Ultimately, professional networks are valuable resources for finding languishing data hidden in field notebooks, filing cabinets, old computers and forgotten project materials. As not all the information we need is in the form of research data [32], metadata, grey literature and other auxiliary sources may also be important. Additionally, movements for open data and transparency can help bring hidden data to light. Therefore, data rescue is embedded in a context of community building from the outset, in a positive feedback loop of outcomes: good sharing practices lead to more findable datasets and increased reuse.

**Figure 1.**
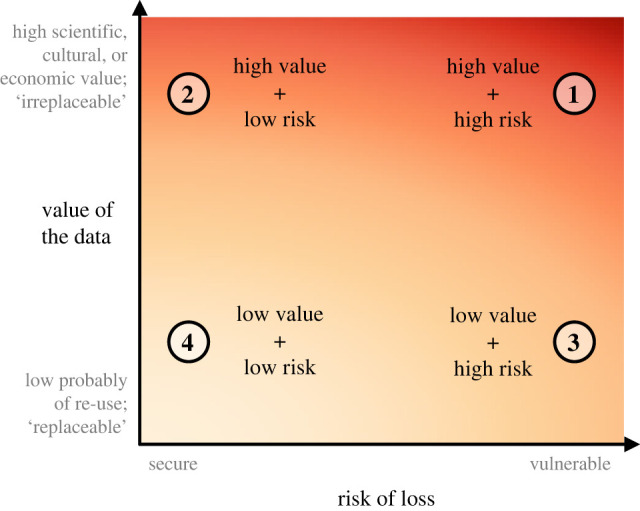
Prioritizing data for rescue: balancing the value of the data and its risk of loss. With many datasets in need of preservation and limited resources, the first step in the data rescue process requires developing a list of priorities for consideration and identifying relevant datasets (figure 2). We consider data prioritization to be a balance between the assessed value of a dataset in question and the potential risk of its loss in the absence of intervention (see *Data prioritization* under *Guidelines*). Alt-text is available in the electronic supplementary material. (Online version in colour.)

**Figure 2.**
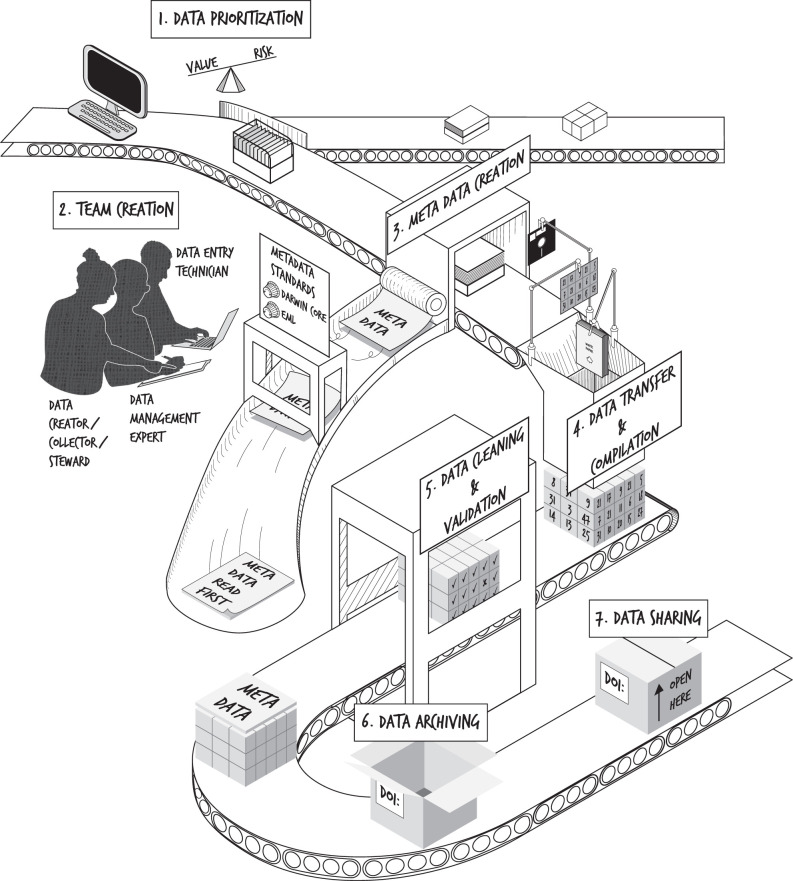
Steps in the data rescue assembly line. First, data must be prioritized for rescue (Step 1). After team creation (Step 2) and metadata creation (Step 3), the data must be transferred and compiled into a logical format (Step 4). After data cleaning and validation (Step 5) is complete, the finalized data and metadata should be archived on a long-term data repository (Step 6). The ultimate goal is to have the rescued data openly available for re-use (Step 7). Alt-text is available in the electronic supplementary material.

Once data has been located, implementing a successful data rescue mission requires a strategic approach (figures 1 and 2). Some elements of data rescue are closely aligned with recommended practices in research data management. Several resources have already outlined ‘best’ practices for data collection [33], management [34] and archiving [4,35–37], yet these are written with current or future data collection in mind and do not address historically collected or unmanaged data. Below, we outline seven steps for data rescue, from identifying high-priority datasets to archiving and sharing them for (re)use.

### (a) Step 1: Data prioritization

Given potentially limited time (and money), some data often need to be prioritized for rescue over others. Prioritizing data for rescue requires consideration along at least two axes: the scientific value of the data and the potential risk that the data will be lost (figure 1). In cases where data are of high value and at high risk, they should be given highest priority. Prioritization becomes less obvious when data rank highly along just one of the axes of value and risk. In such instances, we suggest the focus should be on the value of the data, followed secondarily by risk (i.e. high-value, low-risk data should be prioritized over data that may be at high risk of loss but of low value). The concepts of value and risk of loss are naturally subjective, and myriad factors (e.g. the interests of the rescuer or organization, the combination of datasets to be compared) will impact how value and risk are assessed in each situation. As such, it is challenging to offer objectively clear guidelines for prioritization. There are, however, general characteristics to consider when determining the value and risk of loss of a dataset.

High-value environmental datasets have some common features. Scale is a key factor as datasets comprising long time series or a broad spatial extent are important for establishing temporal and spatial dynamics of change (e.g. population declines, range shifts, etc.). The age of a dataset may be relevant, as older datasets can establish important baselines for a species or system, and the value of such datasets increases with time. The subject of the data is also critical, as their societal value may be greater when involving species or ecosystems with conservation, cultural or economic importance. Additional considerations include the rarity of the data (e.g. data from undersampled regions or ecosystems), uniqueness or irreplaceability (e.g. data from historical events, such as natural disasters) and the potential costs of recollection. Finally, potential future reuse is worth considering, with the highest value datasets having many, immediate potential use scenarios.

The risks of data loss are similarly multifold. Data can be physically lost, especially if there is only one copy (paper or digital). Data can be functionally lost when the datasets are unreadable owing to defunct file formats (e.g. Lotus 1-2-3™) or obsolete storage media (e.g. floppy disks). Data can also be functionally lost when vital knowledge about collection or meaning is lost (e.g. because the collector/creator of the data is deceased, retired or otherwise no longer active in their field). Ultimately, balancing the data's value and risk of loss is essential for effective prioritization of data rescue efforts.

### (b) Step 2: Team creation

Data rescue takes a team, with different roles needed at different points in the rescue process. We first consider those currently in possession of the data, who we collectively refer to as *data custodians*. These include:

(i) *data creators,* who are typically involved in generating the ideas that lead to the data's collection and retain the intellectual property rights and responsibilities for the data;
(ii) *data collectors*, who generate or collect the original data and, therefore, provide valuable input for documenting the data; and
(iii) *data stewards*, who are responsible for managing and maintaining the data (i.e. organizing and keeping data archived, including instances where researchers have been bequeathed data or organizations act as guardians of data collected by past employees).

These roles are often played by the same person, though not always. For example, a graduate student may play all three roles as data creator, collector and (temporary) steward, while the advisor may retain the data long term as the principal investigator, thereby acting as data creator and (long-term) steward. Having at least one person who is a data creator, collector or steward as part of the data rescue team is imperative for a successful data rescue mission.

A *data management expert* is another key role. Usually, a data manager plans the data lifecycle, but in a data rescue project this role is focused on organizing and documenting the digitized datasets. This person will have the skills to connect datasets, clean and manage data, and compile previously unwritten information. Additionally, if any data are not in digital formats, a *data entry technician* will be an integral part of the team, ensuring all necessary data have been digitized in the appropriate format and validated against the original records.

### (c) Step 3: Metadata creation

*Metadata* are information about the data, typically contained in a file separate from the dataset [38]. Metadata describe the data collection process (e.g. types of data collected, methodology and contributors), variables in the dataset (e.g. column headings for tabular data; ‘data dictionary’), abbreviations, units of measurement and other relevant information necessary to understanding how the data were generated and how to (re)use them (e.g. why some measurements are lacking; [34]). We recommend early creation of the metadata, as this often informs the remaining process and structure of the compiled dataset.

For datasets with more than one associated file, the metadata should also include a description of which data are contained in each file and how files are related. For datasets which include ongoing data collection, detailed metadata files are important to ensure that subsequently inputted data conform to existing standards and structure [39]. The metadata should be revised throughout the subsequent steps to incorporate details about the data rescue process (e.g. data manipulation, validation or changes to database structure; figure 2).

Metadata file formats vary, often based on the type of data or chosen repository. In ecology, metadata are often provided in a 'README' style text file that is, at a minimum, ‘human-readable’ (i.e. a person can interpret the information contained in the file). Ideally, metadata should also be ‘machine-actionable’, allowing computers to process and integrate datasets in an automated fashion (*Interoperability*, the third FAIR principle) [25], enabling interaction with large volumes of data—a task that is not possible for humans to do.

A common format for creating metadata that are human- and machine-readable is a text file written in Extensible Markup Language (XML; for basic principles and examples, see https://www.xmlfiles.com/xml). A variation on XML called the Ecological Metadata Language (EML) is a set of suggested 'tags' (variables) to create machine-actionable metadata in ecology (see https://eml.ecoinformatics.org; [40,41]).

A recent alternative to XML is the use of schemas. For example, schema.org (https://schema.org) provides a collection of shared vocabularies to markup data in a standard fashion, allowing them to be understood by major search engines. The schema.org vocabulary is used in combination with a data-interchange language, such as JSON-LD, to structure and add information to data. Guidelines and examples of scientific use of schema.org are available from the Federation of Earth Science Information (https://wiki.esipfed.org/Main_Page) and Bioschemas (https://bioschemas.org). Tools also exist to help ecologists generate a schema and translate it to EML [42].

### (d) Step 4: Data transfer and compilation

For effective collaboration, all team members should have access to the data and metadata files. However, this might only be possible if all files are already in a digital format; any physical copies should first be photographed or scanned, or entrusted to the team member responsible for data entry and validation. While the details of data compilation will need to be tailored to each dataset, the workflow should be as reproducible as possible. For example, any edits made to the data should be done in a file separate from the original; a digital file with untouched original data should always remain. All major decisions should be documented in the metadata.

In structuring the data, we recommend Wickham's [43] tidy data principles (also called 'third normal form' in relational data design [44]), which consist of three core concepts: (i) each variable has its own column, (ii) each observation has its own row, and (iii) each type of observational unit is in its own data table (e.g. individual-level measurements from a population, such as mass, in one table and population-level metrics, such as abundance, in another). If there are multiple data tables, they should be connected to each other by one or more variables that uniquely identify individual observations (i.e. primary keys in a relational database; [44]). While we advocate for tidy data principles, as they are most likely to generate a data structure that will be useful in subsequent analyses, sometimes alternative data structures will be preferred, such as site-by-species matrices for community-level data. Additionally, not all environmental data will be easily represented in tabular form, such as geospatial data or images, though other relevant standards may apply (see below). Finally, note that many data types are not well suited to a relational database model and may benefit from other, equally valid frameworks (e.g. tree/graph-based data models in JSON).

### (e) Step 5: Data cleaning and validation

Data cleaning consists of identifying and fixing issues and can be one of the most time-intensive steps. In addition to correcting typographical or entry errors, data cleaning includes checking for data completeness (i.e. all records are fully transcribed) and uniformity (i.e. variables and units are consistent). The International Organization for Standardization (ISO) provides standards for many common variables such as date-times (ISO 8601) and geographical coordinates (ISO 6709), and many tools exist to help with specific aspects of data cleaning (e.g. the *taxize* R package to check taxonomies; [45]).

Data validation involves the comparison of the dataset against a set of assertions. This is important for ensuring data quality and integrity by confirming that the structure and content of the data are appropriate. In data rescue, unlike most recently or currently collected data, data validation may come with the extra challenge that the original data custodians may be unavailable. As such, having as many original members of the data team as possible is particularly beneficial (see *Team creation*). Common data validation techniques include plotting the data to identify incorrect or improbable values, checking that the contents or dimensions of the data match expectations, cross-checking data from different columns or tables for mutual compatibility and evaluating summary statistics or other outputs that characterize the data. In addition, many tools exist to help with the data validation process, including open-source, 'point-and-click' software (e.g. OpenRefine) and programming tools (e.g. the *assertr* and *validate* R packages; [46,47]).

Although the exact data cleaning and validation steps will vary by dataset, many of the principles described in the *Data transfer and compilation* section are also relevant. Validation should be conducted as reproducibly as possible, and any errors should be corrected without manipulating the original (raw) files. Any changes should be well documented (e.g. as comments in the script or as notes in the metadata), as should the rationale behind the corrections.

Data custodians may also consider providing a checksum (e.g. md5) or cryptographic hash (e.g. SHA-256) for each data file. Checksums and hashes are unique alpha-numeric signatures generated by an algorithm using the reference file as input information, such that even a trivial change in the contents or structure of the file will result in the production of a completely different output. A future potential user (including the original data creator) can then recalculate the hash upon accessing the archived data (see *Data archiving* and *Data sharing*), compare it to the value stored in the metadata and ensure data integrity prior to re-use.

### (f) Step 6: Data archiving

Archiving data in non-proprietary formats is imperative for longevity and future accessibility. Non-proprietary formats are those which do not have a copyright or trademark and, therefore, are part of the public domain. Using non-proprietary formats ensures that anyone can access the data without needing specific software or in the event that the program becomes defunct. For example, tabular data should be stored in comma-separated values (.csv) format or text files (.txt) rather than proprietary formats such as Microsoft Excel^®^ files (.xlsx). More recently, other open-source formats such as Apache parquet files (.parquet) have been developed, enabling highly efficient and compressed storage of 'big' data. Unlike CSVs, parquet files also have the advantage of storing the schema (i.e. column/variable types; see *Metadata creation*) directly in the file metadata, reducing the chance that variables are incorrectly stored or used.

There is a growing movement to archive data on public data repositories rather than, or in addition to, private or institutional systems (e.g. laboratory hard drives). Many governments and funding agencies have recently implemented new data management protocols that encourage or mandate the archiving, though not necessarily sharing, of all data generated using their resources (see below; e.g. Canada's Tri-agency Research Data Management Policy). Each year following publication, data that have not been publicly archived are 17% less likely to be recoverable [5] (see also [48]). As such, we consider public archiving to be an essential part of data rescue, since private archiving does not mitigate the possibility that data will need to be 're-rescued' in the future. Cleaned data and metadata should be placed in a repository, maintaining them in a secure and retrievable format. Importantly, the push for public archiving does not contradict the need for privacy or sensitivity associated with some datasets; it is possible to publicly archive data while maintaining restrictions on when and how the data are accessed. We suggest, however, that most environmental data should be openly accessible upon archiving, with some clear exceptions (e.g. data pertaining to threatened species or Indigenous data sovereignty; see below).

There are many data repositories from which to choose (see https://www.re3data.org/ for a comprehensive list), with some being generalized (e.g. Dryad, Dataverse, Figshare and Zenodo) and others more specified (e.g. DataONE for environmental data, GenBank for genetic sequences). Data repositories tend to use a distributed, decentralized approach to storing data and have contingency plans to ensure the longevity of archived datasets. Choice of repository will be influenced by whether the data will remain private or be made openly accessible upon upload, or soon thereafter [12]. Some repositories allow for long-term storage regardless of whether data are made openly available (e.g. Dataverse), while others mandate open access (e.g. Dryad). Many archives also offer an option to place an embargo on the publication of data. Most data repositories establish a digital object identifier (DOI), a unique identifier which remains constant for the lifetime of the object, even if the object or metadata change. For open data, we suggest explicitly stating the terms of use, such as whether authors should be contacted if the data are to be included in a publication, or adding a copyright statement, such as those from Creative Commons (e.g. CC0, CC-BY, etc.).

### (g) Step 7: Data sharing

The final step in the data rescue workflow is ensuring the data meet open science standards. Open science principles include transparency, participation and accessibility. These values can be addressed in different ways, sometimes making the process overwhelming for researchers who are not trained in data management. The FAIR and CARE principles, the first of which focuses on how data can be made useful and the second on how we can promote justice through responsibly sharing open data, summarize ways these values can be met through a combination of actions.

The **FAIR** principles aim to improve **F**indability, **A**ccessibility, **I**nteroperability and **R**eusability of datasets [25]. Providing human- and machine-readable metadata improves both the findability and accessibility of a dataset. Combined with proper archiving and identification, strong metadata helps increase the discoverability of datasets. As mentioned in the *Data archiving* section, adding a DOI makes the data trackable and citable. A comprehensive metadata file enables interoperability, or the ability of the data to be combined with other datasets in different ways and in different systems. Additionally, accessibility and reusability can be achieved through licences, which explicitly describe the usage and attribution rights of the data.

The **CARE** principles focus on datasets that used traditional knowledge or benefited somehow from Indigenous lands, promoting transparency and participation of open data ([49]; see also, the OCAP principles: https://fnigc.ca/ocap-training/). They aim to address consideration of the **C**ollective benefit for Indigenous Peoples, **A**uthority to control (recognizing Indigenous data sovereignty), **R**esponsibility to be respectful with Indigenous Peoples involved in the dataset collection and **E**thics (by assuring the participation of Indigenous Peoples in the assessment of benefits, harms and usability of the data; [49]). These principles begin to address the larger, complicated history of colonialism in ecology, evolution and related disciplines. While these guidelines were written with current and future data collection in mind, they are equally applicable to and important for previously collected data.

Carroll *et al*. [50] provide valuable guidance on reconciling CARE and FAIR principles with Indigenous data sovereignty at the forefront. Providing specific recommendations for addressing CARE principles in data rescue is challenging and beyond the scope of this paper; each project brings unique circumstances that are best navigated by the data custodians and Indigenous partners. In an ideal scenario, the data creator has established collaborations with relevant Indigenous communities, leading the data rescue effort to become another meaningful collaboration, collectively adjusting the data rescue workflow to address both FAIR and CARE principles—which, as Carroll *et al*. [50] note, need not be in conflict. A full realization of CARE principles would see Indigenous partners oversee data archiving and stewardship, with direct control over access to the repository [50]. Existing tools such as embargo periods (i.e. the delayed release of data) or controlled access (i.e. data hosted on a repository and available by request) may be useful in addressing concerns around sovereignty over sensitive data [15]. In cases where the data custodian has limited experience engaging with Indigenous communities, the potential to achieve CARE principles will depend upon the feasibility of developing trust and respectful relationships with the relevant Indigenous partners. Given the devastating legacies of colonialism, this can take considerable time. Nevertheless, it would rarely be a misstep to request a meeting with local communities to communicate the goals of the data rescue project, highlighting the aim of achieving CARE principles in partnership with the community.

## 3. Conclusion

Ultimately, we hope to reach a point where data rescue is no longer needed. This requires researchers, funding agencies, and publishers to align their views around ethical and professional obligations to publicly archive data as well as a culture change that sees best practices in managing, archiving and sharing data become the default in publicly funded research. To achieve this goal, data sharing and accessibility need to be prioritized as critical components of the scientific enterprise. First, there must be continued, long-term investment in data management [51]. Such investment includes not only infrastructure but also training and support for students and personnel [4,22]. Additionally, publishers, employers and funding agencies must require accountability from researchers to preserve data in accessible formats and, if appropriate, make the data openly available [51]. Until these institutional-level paradigm shifts occur, smaller scale and innovative data rescue is integral to environmental data curation.

Currently, training in data management and shifting regulations regarding data availability has focused on present and future data. With such a strong eye to the future, however, data of the past is being left behind. Data rescue presents an opportunity to mitigate this loss of historical data while also providing additional, less tangible benefits. In the CIEE Living Data Project, our mission of breathing life into languishing data is concomitant with training the next generations of scientists in data management best practices and forging connections among researchers across a wide variety of career stages and trajectories, thus ensuring the longevity of scientific knowledge and preparing students for a data-rich future.

## Data Availability

Electronic supplementary material is available online [52].
